# Analysis of risk factors in breast cancer patients with hand-foot syndrome and oral mucositis caused by pegylated liposomal doxorubicin

**DOI:** 10.3389/fonc.2025.1564681

**Published:** 2025-05-22

**Authors:** Xianghua Quan, Jing Li, Jialin Sun, Xiaomin Xing, Donghua Liu, Hongyan Ji, Qie Guo

**Affiliations:** Department of Clinical Pharmacy, The Affiliated Hospital of Qingdao University, Qingdao, Shandong, China

**Keywords:** adverse reactions, breast cancer, pegylated liposomal doxorubicin, hand-foot syndrome, oral mucositis

## Abstract

**Introduction:**

Doxorubicin (DOX) is a primary treatment for breast cancer (BC), but its widespread use is hindered by cardiotoxicity. Pegylated liposomal doxorubicin (PLD) has been developed to enhance the efficacy of DOX and mitigate its cardiotoxic effects. However, PLD is associated with adverse reactions (ADRs) such as hand-foot syndrome (HFS) and oral mucositis (OM), which have garnered significant attention. Although not life-threatening, HFS and OM can cause severe discomfort and functional impairment. Severe cases may necessitate a reduced PLD dose or even delay or interrupt chemotherapy, ultimately leading to decreased medication compliance. Here, we conduct an analysis of the risk factors associated with HFS and OM during the PLD chemotherapy regimen, thus providing early warning indicators for the potential occurrence of these adverse reactions in BC patients.

**Methods:**

In this study, a total of 395 BC patients receiving PLD chemotherapy were enrolled. Follow-up observations towards the baseline and clinical characteristics in these patients were exhibited. The evaluation of HFS and OM in these patients was also performed based on the Common Terminology Criteria for Adverse Events (CTCAE) Version 5.0. Analysis of factors influencing simultaneous incidence of HFS and OM was executed using the univariate analysis and multivariate logistic regression analysis.

**Results:**

Dose intensity, history of cholelithiasis, ALT, AST, and Hb were identified as related risk factors for HFS. Dose intensity and reductions in white blood cell (WBC) counts were associated with the risk of OM. Furthermore, increased dose intensity, decreased WBC counts, and a history of cholelithiasis emerged as independent risk factors for the concurrent occurrence of HFS and OM.

**Discussion:**

This study investigated the various risk factors related to HFS, OM, and their combination in BC patients undergoing PLD chemotherapy, offering insights for the prevention and treatment of BC and other cancers.

## Introduction

Breast cancer (BC) accounts for one-quarter of cancer cases and one-sixth of cancer deaths among women (1, 2). Doxorubicin (DOX)-based systemic chemotherapy is the first line of treatment for BC (3). Pegylated liposomal doxorubicin (PLD) consists of a DOX core encapsulated within a phospholipid microcapsule, with an outer layer modified by hydrophilic polyethylene glycol (4). This unique structure, including membrane composition, physical state, internal environment, particle size, surface charge, and structural modifications, can enhance both the permeation and retention effects. As a result, PLD exhibits excellent targeting properties, accumulates at high concentrations in tumor tissues, and has strong anti-tumor effects (5). However, treatment with PLD can readily lead to hand-foot syndrome (HFS) and oral mucositis (OM). HFS presents as erythema, abnormal swelling, or a tingling sensation on the palms, soles, and toes, potentially progressing to blistering, desquamation, ulceration, erosion, and epidermal necrosis in severe cases (6). The incidence rate of PLD-induced HFS is 29%-50% (7, 8), which may occur as early as 3-5 days after the initial treatment, and generally in the 2nd-3rd cycle of the treatment. OM is a common oral inflammatory side effect of chemotherapy with PLD, with an incidence rate of 10%-68% (9).OM typically appears within 4-7 d after the initiation of chemotherapy, peaking in severity between 10-14 days (10). It manifests as varying degrees of oral mucosal edema, erythema, ulceration, erosion, and secondary infections. Patients may experience dry mouth, localized pain, difficulty eating, and taste disorders (11, 12). This study retrospectively analyzed the factors associated with the occurrence of HFS and OM, aiming to provide a theoretical basis for the prevention of these conditions and the individualized treatment of PLD.

## Materials and methods

### Subjects

**Figure 1:**
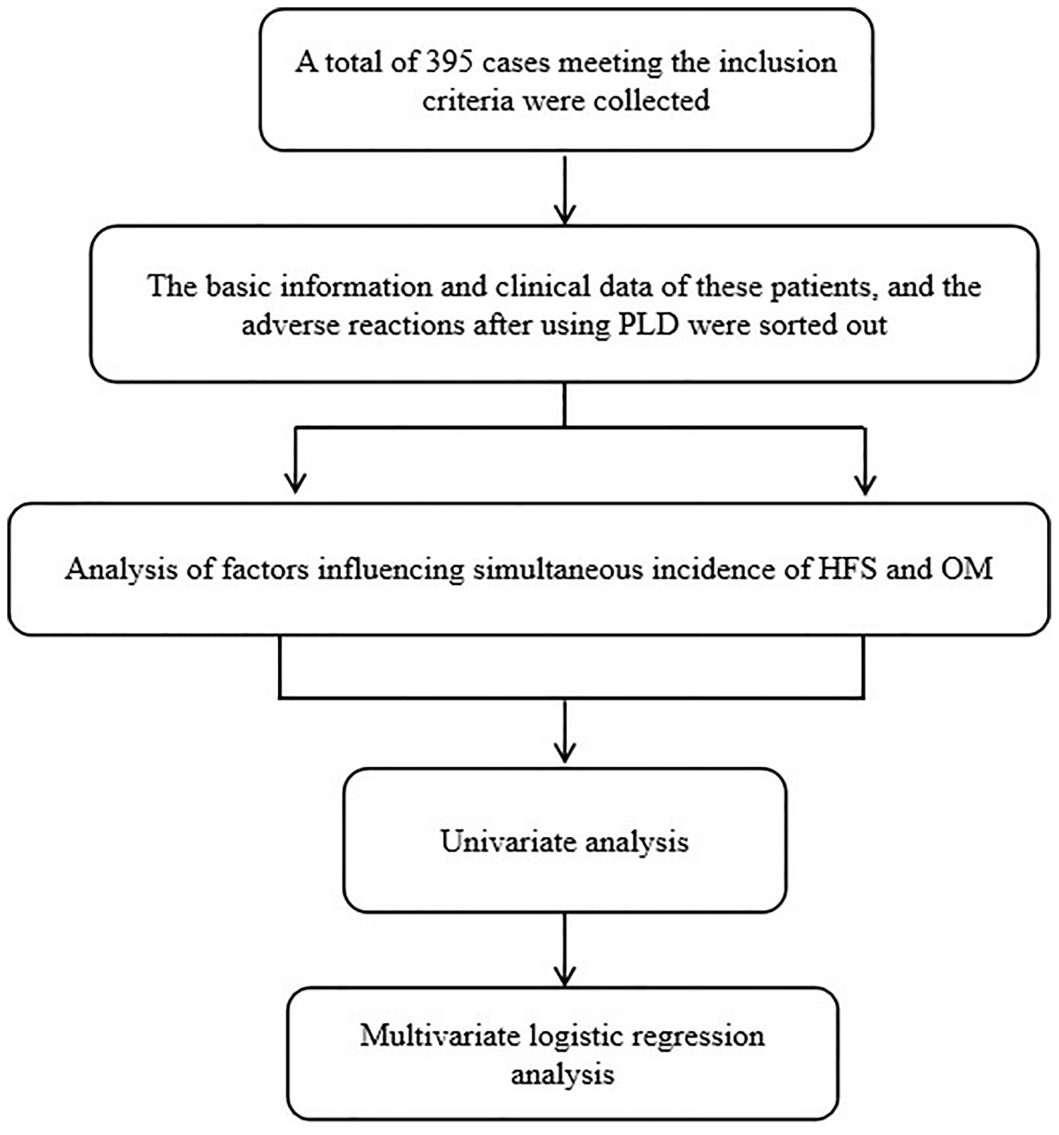
The flow chart of this study.

A total of 437 BC patients who received PLD chemotherapy from October 2019 to December 2021 in a breast diagnosis and treatment center of the Affiliated Hospital of Qingdao University were preliminary collected. Subsequently, a total of 395 female patients with BC aging over 18 years old were enrolled according to inclusion and exclusion criteria. These patients have underwent chemotherapy for the first time with PLD regime including AC-T(PLD/Cyclophosphamide-Paclitaxel),TAC (Docetaxel/PLD/cyclophosphamide) and AT (Docetaxel/PLD). The dose intensity of PLD is either 30 or 35 mg/m2. The patients who were diagnosed as other malignant tumors within 5 years and exhibited metastatic lesions were excluded. Those patients that were allergic to DOX or PLD with incomplete case data, and were previously diagnosed with history of skin diseases, hand and foot diseases, or oral diseases were excluded. This study was approved by the ethics committee of the Affiliated Hospital of Qingdao University. Informed written consent was obtained from all patients. The work flow of this study is as follows in **Figure 1**.

The basic information and clinical data of these patients were collected, and the adverse reactions (ADRs) after PLD administration were sorted out. Furthermore, the baseline and clinical characteristics were compared according to whether the patients had HFS and OM. The risk factors for HFS and OM occurrence were also discussed by multivariate Logistic regression analysis.

### The evaluation of ADRs in patients with PLD-based chemotherapy

The ADRs that patients experience during chemotherapy were assessed and graded according to the Common Terminology Criteria for Adverse Events (CTCAE) Version 5.0 (13).The details were shown in **Tables 1**, **2**.

**Table 1:** Classification standards of HFS.

Grade	Clinical Manifestations
**Grade I**	Painless minor skin changes or dermatitis such as erythema, edema, or excessive keratinization)
**Grade II**	Painful skin changes such as peeling, blistering, bleeding, cracking, edema or, excessive keratinization
**Grade III**	Severe skin changes (e.g., peeling, blistering, bleeding, cracking, edema, hyperkeratosis)

**Table 2:** Classification standards of OM.

	Grade I	Grade II	Grade III	Grade IV
Clinical manifestations	Asymptomatic or mild symptoms	Moderate pain or ulcers that does not affect oral feeding	Severe pain that affects oral feeding	Endangering life and Urgent treatment required

### Follow-up observations

Follow-up visits were performed and the following observations were executed:

1. General information including Age, BMI, BSA, ECOG score and pathological type.
2. Previous medical history including hypertension, diabetes, gallstone and hepatic cyst.
3. Chemotherapy data including dose intensity of PLD and chemotherapy regimen.
4. The determination of baseline characteristic including ALT, AST, GGT, TBIL, Cr, MONO, WBC, NEUT,PLT, and Hb.
5. The expression of pathological indicators including ER, PR, HER-2, and Ki-67.

Clinicopathological features for these BC patients were shown in **Table 3**. Here, we expected to be able to provide early warning of the occurrence of HFS and OM by the baseline measures of patients before treatment. The risk assessment of HFS and OM will lag if the indicators after treatment are evaluated or the corresponding indicators are different before and after. Therefore, baseline values of ALT, AST, GGT, TBIL, Cr, MONO, WBC, NEUT, PLT, Hb etc. before treatment were included in statistical analysis to further evaluate their correlation with HFS and OM onset.

**Table 3:** Clinicopathological features of BC patients.

Clinical features		N(%)/ $\bar{X}$ ± S/M(Q25-Q75)
Age		50.44 ± 9.10
BMI		
	<25	171 (43.3%)
	≥25	224 (56.7%)
ECOG score		
	0	357 (90.4%)
	≥1	38 (9.6%)
Chemotherapy dose intensity		
	30	187 (47.3%)
	35	208 (52.7%)
Chemotherapy regimens		
	AC-T	363 (91.9%)
	TAC	14 (3.5%)
	TA	18 (4.6%)
Pathological type		
	HER-2^+^(HR^+^)	55 (13.9%)
	HER-2^+^(HR^-^)	49 (12.4%)
	TNBC	89 (22.5%)
	Luminal A	150 (38.0%)
	Luminal B	52 (13.2%)
Previous diseases		
	Hypertension	48 (12.2%)
	Diabetes	17 (4.3%)
	Gallstone	8 (2.0%)
	Liver cyst	5 (1.3%)
ER		
	–	137 (34.7%)
	+	258 (65.3%)
PR		
	–	153 (38.7%)
	+	242 (61.3%)
Ki-67		
	Low	105 (26.6%)
	High	290 (73.4%)
HER2		
	–	293 (74.2%)
	+	102 (25.8%)
Baseline ALT		35 (22-55)
Baseline AST		30 (23-40)
Baseline GGT		16 (11-23)
Baseline TBIL		11.60 (9.10-14.80)
Baseline Cr		56 (49-70)
Baseline MONO		0.6 (0.5-0.7)
Baseline WBC		4.2 (3.4-5.3)
Baseline NEUT		2.5 (1.9-3.4)
Baseline PLT		254 (213-298)
Baseline Hb		126 (118-134)

BMI, body mass index; normal BMI (<25.0 kg/m2) and overweight status (≥25.0 kg/m2); ECOG, Eastern Cooperative Oncology Group; ER , Estrogen receptor; PR, Progesterone Receptor; HER2, Human epidermal growth factor receptor 2; ALT, Alanine aminotransferase; AST, Aspartate aminotransferase; GGT, Gama-glutamyltransferase; TBIL, Total bilirubin; Cr, Creatinine; MONO, Monocytes; WBC, White blood cell; NEUT, Neutrophils; PLT, Platelet; Hb, Hemoglobin.

### Statistical analysis

Statistical analysis was performed using SPSS 25.0. Measurement data that conform to normal distribution are represented as $\bar{X}$ ± S. Counting data analysis was performed using χ2 test, t-test and non parametric rank sum test. Multivariate logistic regression analysis was performed to determine the factors related with the occurrence of HFS or/and OM.

## Results

### Severity level of HFS and disease cycle

**Figure 2:**
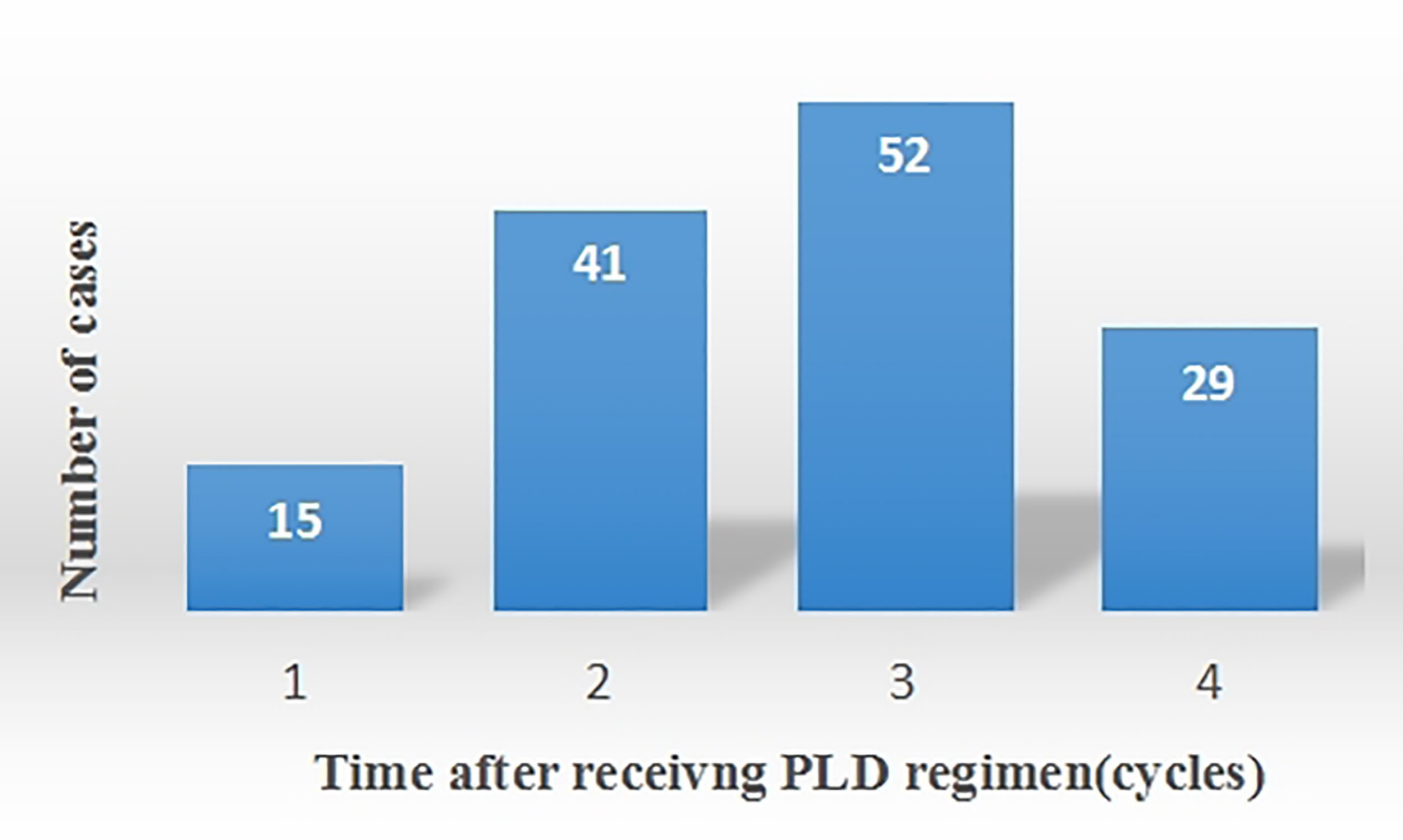
The occurrence of HFS after receiving PLD regimen.

The ADRs that patients experience during chemotherapy were assessed (**Table 4**). 137 cases of HFS were observed. Among these, 55 cases were classified as Grade I HFS, 59 as Grade II, and 23 as Grade III (**Table 5**). HFS developed in 15 patients after the 1st cycle of chemotherapy, 41 patients after the 2nd cycle, 52 patients after the 3rd cycle, and 29 patients after the 4th cycle (**Figure 2**). Consequently, HFS predominantly occurred after 2-3 cycles of chemotherapy, accounting for 67.9% of the total HFS cases.

**Table 4:** Summary of ADRs in BC patients with PLD treatment.

Adverse reactions		Number (%)	Grade I∼II(%)	Grade III∼IV (%)
HFS		137 (34.7%)	114 (28.9%)	23 (5.8%)
OM		55 (13.9%)	45 (11.4%)	10 (2.5%)
Gastrointestinal disorders	Nausea	59 (14.9%)	57 (14.4%)	2 (0.5%)
	Vomiting	77 (19.5%)	70 (17.7%)	7 (1.8%)
	Diarrhea	8 (2.0%)	7 (1.8%)	1 (0.3%)
	Abdominal pain	5 (1.3%)	5 (1.3%)	0
	Astriction	7 (1.8%)	7 (1.8%)	0
Respiratory damage				
	Cough	3 (0.8%)	3 (0.8%)	0
Neurological damage				
	Dizzy	4 (1.0%)	4 (1.0%)	0
	Headache	3 (0.8%)	3 (0.8%)	0
	Agrypnia	10 (2.5%)	10 (2.5%)	0
Hematologic disorders				
	NEUT reduction	243 (61.5%)	200 (50.6%)	43 (10.9%)
	WBC reduction	220 (55.7%)	182 (46.1%)	38 (9.6%)
	PLT reduction	15 (3.8%)	14 (3.5%)	1 (0.3%)
	Hb reduction	133 (33.7%)	126 (32.5%)	7 (1.8%)
Systemic system				
	Local irritant effect	5 (1.3%)	5 (1.3%)	0
	Feeble	7 (1.8%)	7 (1.8%)	0
Cardiac dysfunction				
	Cardiotoxicity	14 (3.5%)	14 (3.5%)	0
Eye toxicity				
	Xerophthalmia	3 (0.8%)	3 (0.8%)	0
	Conjunctivitis	5 (1.3%)	5 (1.3%)	0
Hepatic dysfunction				
	ALT increase	150 (38.0%)	144 (36.5%)	7 (1.8%)
	AST increase	153 (38.7%)	150 (38.0%)	3 (0.8%)
	GGT increase	40 (10.1%)	38 (9.6%)	2 (0.5%)
	TBIL increase	20 (5.1%)	19 (4.8%)	1 (0.3%)

HFS, Hand-foot syndrome; OM, Oral mucositis; ALT, Alanine aminotransferase; AST, Aspartate aminotransferase; GGT, Gama-glutamyltransferase; TBIL , Total bilirubin; WBC, White blood cell; NEUT, Neutrophils; PLT, Platelet; Hb, Hemoglobin.

**Table 5:** Summary of HFS in BC patients with PLD treatment.

	Grade I	Grade II	Grade III	Total
Number of cases	55	59	23	137

### Severity level of OM and disease cycle

**Figure 3:**
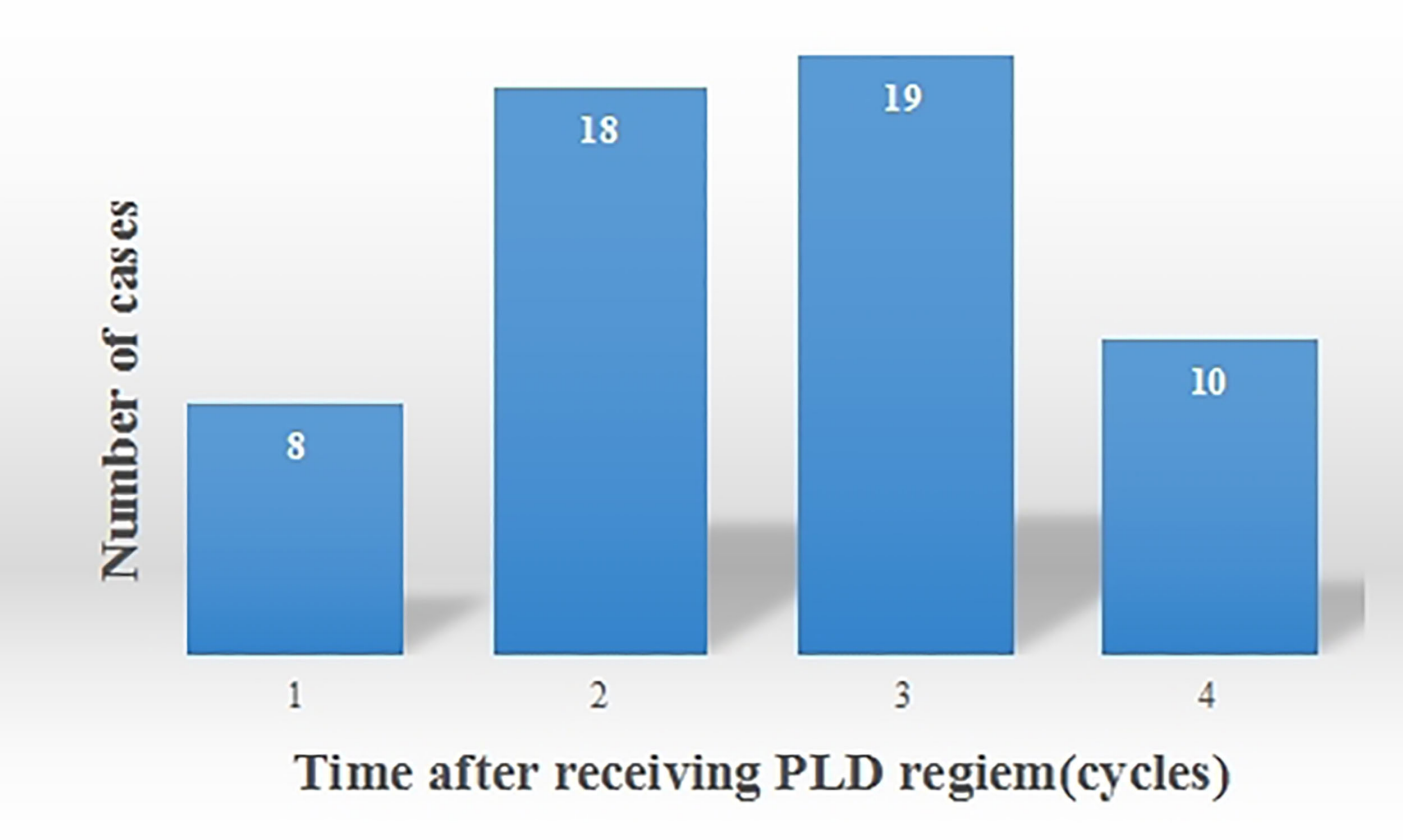
The occurrence of OM after receiving PLD regimen.

In this study, 55 cases of OM were noted. Among these, 16 cases were classified as Grade I OM, 29 as Grade II, and 10 as Grade III (**Table 6**). OM developed in 8 patients after the 1st cycle of chemotherapy, 18 patients after the 2nd cycle, 19 patients after the 3rd cycle, and 10 patients after the 4th cycle (**Figure 3**). Therefore, OM predominantly occurred after 2-3 cycles of chemotherapy, accounting for 67.3% of the total OM cases.

**Table 6:** Summary of OM in BC patients with PLD treatment.

	Grade I	Grade II	Grade III	Grade IV	Total
Number of cases	16	29	10	0	55

### Univariate analysis of HFS

Among the 395 BC cases enrolled, 137 patients suffered from HFS while 258 were not. Clinical data analysis revealed that the incidence rate of HFS was 45.7% (95/208) under a dose intensity of 35 mg/m² and 22.5% (42/187) under a dose intensity of 30 mg/m² (P<0.001). Additional significant factors included a history of cholelithiasis (P=0.002), hepatic cyst (P=0.032), and elevated liver enzymes. The median ALT levels were 48 U/L in the HFS group compared to 28 U/L in the HFS-f2ree group (P<0.001), the median AST levels were 38 U/L versus 26 U/L (P<0.001), and the median GGT levels were 17 U/L versus 15 U/L (P=0.001). Additionally, the median hemoglobin (Hb) levels were 123 g/L in the HFS group and 128 g/L in the HFS-free group (P=0.007). These differences were statistically significant. However, there were no significant differences in age, BMI, ECOG performance status, chemotherapy regimen, molecular typing, histological grading, hypertension, diabetes, ER, PR, Ki-67, HER-2, TBIL, Cr, MONO, WBC, NEUT, and PLT. The details for the clinical features between HFS group and non-HFS group were demonstrated in **Table 7**.

**Table 7:** Comparison of clinical features between HFS group and non-HFS group.

Clinical features	HFS group (137)	Non-HFS group (258)	*P value*
Age	50.66±9.865	50.33±8.64	0.730
BMI			0.954
<25	79 (57.7)	148 (57.4)	
≥25	58 (42.3)	110 (42.6)	
ECOG score			0.155
0	120 (87.6)	238 (92.2)	
1	17 (12.4)	20 (7.8)	
Chemotherapy dose intensity (mg·m^2^)			<0.001^*^
30	42 (30.7)	145 (56.2)	
35	95 (69.3)	113 (43.8)	
Chemotherapy regimens			0.060
AC-T	122 (89.0)	241 (93.4)	
TAC	9 (6.6)	5 (1.9)	
AT	6 (4.4)	12 (4.7)	
Pathological type			0.181
HER-2^+^(HR^+^)	14 (10.2)	41 (15.9)	
HER-2^+^(HR^-^)	18 (13.1)	31 (12.0)	
TNBC	32 (23.4)	57 (22.1)	
Luminal A	60 (43.8)	90 (34.9)	
Luminal B	13 (9.5)	39 (15.1)	
Hypertension			0.278
Yes	20 (14.6)	28 (10.9)	
No	117 (85.4)	230 (89.1)	
Diabetes			0.106
Yes	9 (6.6)	8 (3.1)	
No	128 (93.4)	250 (96.9)	
Gallstone			0.002^*^
Yes	7 (5.1)	1 (0.4)	
No	130 (94.9)	257 (99.6)	
Liver cyst			0.032^*^
Yes	4 (2.9)	1 (0.4)	
No	133 (97.1)	257 (99.6)	
ER			0.581
–	50 (36.5)	87(33.7)	
+	87 (63.5)	171 (66.3)	
PR			0.393
–	57 (41.6)	96 (37.2)	
+	80 (58.4)	162 (62.8)	
Ki-67			0.413
Low	33 (24.1)	72 (27.9)	
High	104 (75.9)	186 (72.1)	
HER-2			0.168
–	107 (78.1)	185 (71.7)	
+	30 (21.9)	73 (28.3)	
Baseline ALT	48 (37-71)	28 (18-42)	<0.001^*^
Baseline AST	38 (31-46)	26 (21-35)	<0.001^*^
Baseline GGT	17 (13-28)	15 (11-21)	0.001^*^
Baseline TBIL	11.90 (8.90-14.80)	11.60 (9.20-14.80)	0.980
Baseline Cr	57 (49-72)	55 (48-70)	0.337
Baseline MONO	0.6 (0.5-0.7)	0.6 (0.5-0.7)	0.269
Baseline WBC	4.0 (3.3-5.5)	4.3 (3.4-5.2)	0.265
Baseline NEUT	2.5 (1.8-3.5)	2.5 (2.0-3.4)	0.506
Baseline PLT	256 (211-304)	251 (213-296)	0.606
Baseline Hb	123 (116-133)	128 (119-135)	0.007^*^

BMI, body mass index; ECOG, Eastern Cooperative Oncology Group; ER, Estrogen receptor; PR, Progesterone Receptor; HER2, Human epidermal growth factor receptor 2; ALT, Alanine aminotransferase; AST, Aspartate aminotransferase; GGT, Gama-glutamyltransferase; TBIL, Total bilirubin; Cr, Creatinine; MONO, Monocytes; WBC, White blood cell; NEUT, Neutrophils; PLT, Platelet; Hb, Hemoglobin; AC-T, Doxorubicin/Cyclophosphamide-Paclitaxel; TAC, Docetaxel/Doxorubicin/cyclophosphamide; AT, Docetaxel/Doxorubicin.

Given that elevated ALT/AST along with a history of cholelithiasis, are all related to liver function, which may indicate collinearity or interaction. We further calculated the variance inflation factors (VIFs) for all included variables in **Table 7**. The results indicate that all VIF values are below 2, suggesting that there is no significant multicollinearity issue within the data (**Supplementary Table 1**). Secondly, we employed an interaction model of alanine aminotransferase/aspartate aminotransferase levels alongside a history of cholelithiasis to analyze the factors influencing the occurrence of HFS through utilizing the variables presented in the **Table 7**. These findings suggested that the interaction terms of ALT/AST and history of cholelithiasis were all significant (P*<0.05), indicating that the effect of elevated ALT/AST on the risk of HFS was stronger in patients with cholelithiasis (**Supplementary Table 2**).

### Multivariate logistic regression analysis of factors influencing HFS

Based on the results of univariate analysis, a multivariate logistic regression analysis was conducted with the incidence of HFS as the dependent variable. The independent variables included dose intensity, history of cholelithiasis, hepatic cyst, ALT, AST, GGT, and Hb. The analysis identified dose intensity (P=0.020), history of cholelithiasis (P=0.037), ALT (P=0.035), AST (P=0.016), and Hb (P=0.001) as independent risk factors for the incidence of HFS (**Table 8**). We also performed another multivariate analysis to examine the factors influencing the occurrence of HFS, utilizing an interaction model that incorporates alanine aminotransferase/aspartate aminotransferase levels and a history of cholelithiasis (**Supplementary Table 3**). These findings indicated that the protective effect of ALT on HFS is significantly enhanced in patients suffering from cholelithiasis. Furthermore, cholelithiasis may influence the risk relationship between AST and HFS. Notably, the incidence rate of HFS in patients with a history of cholelithiasis was 10.460 times higher than in those without a history of cholelithiasis.

**Table 8:** A univariate analysis performed on the HFS group.

Parameters	B	S.E	Wald	Sig	Exp (B)	95% confidence interval for EXP (B)	
						Lower	Upper
Dose intensity	0.605	0.260	5.419	0.020	1.831	1.100	3.047
History of gallstones	2.348	1.124	4.363	0.037	10.460	1.156	94.677
History of liver cysts	1.019	1.227	0.689	0.406	2.769	0.250	30.673
Baseline ALT	0.017	0.008	4.451	0.035	1.018	1.001	1.034
Baseline AST	0.038	0.016	5.840	0.016	1.039	1.007	1.071
Baseline GGT	0.008	0.008	0.994	0.319	1.008	0.993	1.023
Baseline Hb	-0.033	0.010	10.806	0.001	0.967	0.948	0.987

ALT, Alanine aminotransferase; AST, Aspartate aminotransferase; GGT, Gama-glutamyltransferase; Hb, Hemoglobin. Hosmer-Lemeshow Test Statistic: 6.419; P=0.601. The p-value (0.601) is greater than the significance level of 0.05, indicating that there is insufficient evidence to reject the null hypothesis. Consequently, it can be concluded that the model fits the data adequately.

### Univariate analysis of factors influencing OM

Among the 395 BC cases enrolled, 55 developed OM while 340 did not. Clinical data analysis revealed that the incidence rate of OM was 19.2% (40/208) under a dose intensity of 35 mg/m² and 8.0% (15/187) under a dose intensity of 30 mg/m² (P=0.001). The median ALT levels were 40 U/L in the OM group compared to 34 U/L in the OM-free group (P=0.012), and the median AST levels were 34 U/L versus 29 U/L, respectively (P=0.002). Additionally, the median WBC count was 3.4×10_9_/L in the OM group compared to 4.3×10_9_/L in the OM-free group (P<0.001), and the median hemoglobin (Hb) levels were 121 g/L in the OM group versus 127 g/L in the OM-free group (P=0.005). These differences were statistically significant. However, there were no significant differences in age, BMI, ECOG performance status, chemotherapy regimen, molecular typing, hypertension, diabetes, history of cholelithiasis, hepatic cyst, ER, PR, Ki-67, HER-2, GGT, TBIL, Cr, MONO, NEUT, and PLT between the two groups. The details for the clinical features between OM group and non-OM group were demonstrated in **Table 9**.

**Table 9:** Comparison of clinical features between OM group and non OM group.

Parameters	OM group (55)	Non-OM group (340)	*P value*
Age	51.58±10.66	50.26±8.80	0.315
BMI			0.908
<25	32 (58.2)	195 (57.4)	
≥25	23 (41.8)	145 (42.6)	
ECOG score			0.155
0	47 (85.5)	311 (91.5)	
1	8 (14.5)	29 (8.5)	
Chemotherapy dose intensity			0.001^*^
30	15 (27.3)	172 (50.6)	
35	40 (72.7)	168 (49.4)	
Chemotherapy regimens			0.676
AC-T	50 (90.9)	313 (92.1)	
TAC	3 (5.5)	11 (3.2)	
AT	2 (3.6)	16 (4.7)	
Pathological type			0.050
HER-2^+^ (HR^+^)	5 (9.1)	50 (14.7)	
HER-2^+^ (HR^-^)	5 (9.1)	44 (12.9)	
TNBC	17 (30.9)	72 (21.2)	
Luminal A	26 (47.3)	124 (36.5)	
Luminal B	2 (3.6)	50 (14.7)	
Hypertension			0.454
Yes	5 (9.1)	43 (12.6)	
No	50 (90.9)	297 (87.4)	
Diabetes			0.650
Yes	3 (5.5)	14 (4.1)	
No	52 (94.5)	326 (95.9)	
Gallstone			0.052
Yes	3 (5.5)	5 (1.5)	
No	52 (94.5)	335 (98.5)	
Liver cyst			0.693
Yes	1 (1.8)	4 (1.2)	
No	54 (98.2)	336 (98.8)	
ER			0.372
–	22 (40.0)	115 (33.8)	
+	33 (60.0)	225 (66.2)	
PR			0.270
–	25 (45.5)	128 (37.6)	
+	30 (54.5)	212 (62.4)	
Ki-67			0.234
Low	11 (20.0)	94 (27.6)	
High	44 (80.0)	246 (72.4)	
HER-2			0.151
–	45 (81.8)	247 (72.6)	
+	10 (18.2)	93 (27.4)	
Baseline ALT	40 (32-61)	34 (21-53)	0.012^*^
Baseline AST	34 (28-45)	29 (22-39)	0.002^*^
Baseline GGT	17 (12-25)	16 (11-23)	0.213
Baseline TBIL	12.39 (9.25-17.55)	11.50(9.10-14.50)	0.083
Baseline Cr	57 (47-74)	56 (49-70)	0.912
Baseline MONO	0.6 (0.5-0.7)	0.6 (0.5-0.7)	0.066
Baseline WBC	3.4 (3.1-4.8)	4.3 (3.5-5.3)	<0.001^*^
Baseline NEUT	2.5 (1.9-3.3)	2.5 (1.9-3.5)	0.921
Baseline PLT	256 (190-300)	254 (214-298)	0.413
Baseline Hb	121 (117-129)	127 (118-135)	0.005^*^

BMI, body mass index; ECOG, Eastern Cooperative Oncology Group; ER, Estrogen receptor; PR, Progesterone Receptor; HER2, Human epidermal growth factor receptor 2; ALT, Alanine aminotransferase; AST, Aspartate aminotransferase; GGT, Gama-glutamyltransferase; TBIL, Total bilirubin; Cr, Creatinine; MONO, Monocytes; WBC, White blood cell; NEUT, Neutrophils; PLT, Platelet; Hb, Hemoglobin; AC-T, Doxorubicin/Cyclophosphamide-Paclitaxel; TAC, Docetaxel/Doxorubicin/cyclophosphamide; AT, Docetaxel/Doxorubicin.

### Multivariate logistic regression analysis of OM

A multivariate logistic regression analysis was conducted with the incidence of OM as the dependent variable. The independent variables included dose intensity, ALT, AST, WBC, and Hb. The analysis identified dose intensity (P=0.004) and WBC (P=0.009) as independent risk factors for the incidence of OM (**Table 10**).

**Table 10:** A univariate analysis performed on the OM group.

Parameters	B	S.E	Wald	Sig	Exp (B)	95% confidence interval for EXP (B)	
						Lower	Upper
Dose intensity	0.948	0.333	8.102	0.004	2.580	1.343	4.955
Baseline ALT	0.002	0.005	0.140	0.709	1.002	0.993	1.011
Baseline AST	0.005	0.005	1.087	0.297	1.005	0.995	1.016
Baseline WBC	-0.332	0.127	6.833	0.009	0.717	0.559	0.920
Baseline Hb	-0.017	0.012	1.947	0.163	0.983	0.961	1.007

ALT, Alanine aminotransferase; AST, Aspartate aminotransferase; WBC, White blood cell; Hb, Hemoglobin.Hosmer-Lemeshow Test Statistic: 5.876; P=0.661. The p-value (0.661) is greater than 0.05, suggesting that the model has a good fit to the data.

### Univariate analysis of factors influencing simultaneous incidence of HFS and OM

In this study, the HFS + OM group consisted of 46 cases, while the remaining 349 cases were divided among other groups-91 in the HFS only group, 9 in the OM only group, and 249 in the control group. The rate of concurrent HFS and OM was 16.0% (34/208) for a dose intensity of 35 mg/m² and 7.3% (12/187) for a dose intensity of 30 mg/m² (P=0.002). Significant factors included molecular typing (P=0.011) and a history of cholelithiasis (P=0.021). Median ALT levels were higher in the HFS + OM group at 41 U/L compared to 33 U/L in other groups (P<0.001), and median AST levels were 38 U/L in the HFS + OM group versus 29 U/L in others (P<0.001). Additionally, the median WBC count was lower in the HFS + OM group at 3.4×10_9_/L compared to 4.3×10_9_/L in other groups (P=0.001), and the median hemoglobin levels were 121 g/L in the HFS + OM group versus 127 g/L in others (P=0.012). These differences were statistically significant. No significant differences were observed in age, BMI, ECOG performance status, chemotherapy regimen, hypertension, diabetes, hepatic cyst, ER, PR, Ki-67, HER-2, GGT, TBIL, Cr, MONO, NEUT, and PLT between the groups (**Table 11**).

**Table 11:** Comparison of clinicopathological features between co-occurring and non-occurring groups.

Parameters	Co-occurring group (46)	Individual and non-occurring group (349)	*P value*
Age	52.24±11.02	50.20±8.77	0.153
BMI			0.620
<25	28 (60.9)	199 (57.0)	
≥25	18 (39.1)	150 (43.0)	
ECOG score			0.147
0	39 (84.8)	319 (91.4)	
1	7 (15.2)	30 (8.6)	
Chemotherapy dose intensity			0.002^*^
30	12 (26.1)	175 (50.1)	
35	34 (73.9)	174 (49.9)	
Chemotherapy regimens			0.950
AC-T	42 (91.4)	321 (92.0)	
TAC	2 (4.3)	12 (3.4)	
AT	2 (4.3)	16 (4.6)	
Pathological type			0.011^*^
HER-2^+^(HR^+^)	3 (6.5)	52 (14.9)	
HER-2^+^(HR^-^)	4 (8.7)	45 (12.9)	
TNBC	17 (36.9)	72 (20.6)	
Luminal A	21 (45.7)	129 (37.0)	
Luminal B	1 (2.2)	51 (14.6)	
Hypertension			0.777
Yes	5 (10.9)	43 (12.3)	
No	41 (89.1)	306 (87.7)	
Diabetes			0.430
Yes	3 (6.5)	14 (4.0)	
No	43 (93.5)	335 (96.0)	
Gallstone			0.021^*^
Yes	3 (6.5)	5 (1.4)	
No	43 (93.5)	344 (98.6)	
Liver cyst			0.558
Yes	1 (2.2)	4 (1.1)	
No	45 (97.8)	345 (98.9)	
ER			0.096
–	21 (45.7)	116 (33.2)	
+	25 (54.3)	233 (66.8)	
PR			0.178
–	22 (47.8)	131 (37.5)	
+	24 (52.2)	218 (62.5)	
Ki-67			0.133
Low	8 (17.4)	97 (27.8)	
High	38 (82.6)	252 (72.2)	
HER-2			0.074
–	39 (84.8)	253 (72.5)	
+	7 (15.2)	96 (27.5)	
Baseline ALT	41 (36-64)	33 (21-53)	<0.001^*^
Baseline AST	38 (31-46)	29 (22-39)	<0.001^*^
Baseline GGT	18 (13-28)	16 (11-23)	0.062
Baseline TBIL	12.24 (9.10-16.40)	11.60 (9.13-14.60)	0.284
Baseline Cr	60 (49-75)	55 (49-70)	0.328
Baseline MONO	0.6 (0.5-0.7)	0.6 (0.5-0.7)	0.168
Baseline WBC	3.4 (3.0-4.8)	4.3 (3.4-5.3)	0.001^*^
Baseline NEUT	2.5 (1.8-3.3)	2.5 (1.9-3.4)	0.890
Baseline PLT	258 (191-305)	252 (214-297)	0.910
Baseline Hb	121 (117-129)	127 (118-135)	0.012^*^

BMI, body mass index; ECOG, Eastern Cooperative Oncology Group; ER, Estrogen receptor; PR, Progesterone Receptor; HER2, Human epidermal growth factor receptor 2; ALT, Alanine aminotransferase; AST, Aspartate aminotransferase; GGT, Gama-glutamyltransferase; TBIL, Total bilirubin; Cr, Creatinine; MONO, Monocytes; WBC, White blood cell; NEUT, Neutrophils; PLT, Platelet; Hb, Hemoglobin; AC-T, Doxorubicin/Cyclophosphamide-Paclitaxel; TAC, Docetaxel/Doxorubicin/cyclophosphamide; AT, Docetaxel/Doxorubicin.

### Multivariate Logistic regression analysis of simultaneous incidence of HFS and OM

A logistic regression analysis was performed using the simultaneous occurrence of HFS and OM as the dependent variable. The independent variables included in the analysis were dose intensity, molecular typing, history of cholelithiasis, ALT, AST, WBC, and Hb. The results indicated that dose intensity (P=0.014), a history of cholelithiasis (P=0.019), and WBC count (P=0.009) were independent risk factors for the concurrent incidence of HFS and OM (**Table 12**).

**Table 12:** Multivariate logistic regression analysis of risk factors for co-occurrence of HFS and OM.

Parameters	B	S.E	Wald	Sig	Exp (B)	95% confidence interval for EXP (B)	
						Lower	Upper
Dose intensity	0.907	0.370	6.012	0.014	2.476	1.200	5.111
Pathological type	0.006	0.138	0.002	0.967	1.006	0.768	1.318
History of gallstones	1.918	0.820	5.465	0.019	6.808	1.363	33.992
Baseline ALT	0.005	0.005	1.067	0.302	1.005	0.996	1.014
Baseline AST	0.006	0.005	1.047	0.306	1.006	0.995	1.016
Baseline WBC	-0.364	0.139	6.858	0.009	0.695	0.530	0.913
Baseline Hb	-0.019	0.013	1.952	0.162	0.982	0.957	1.007

ALT, Alanine aminotransferase; AST, Aspartate aminotransferase; WBC, White blood cell; Hb, Hemoglobin. Hosmer-Lemeshow Test Statistic: 7.243; P=0.512. The p-value of 0.512 exceeds the threshold of 0.05, suggesting that the model demonstrates a strong fit to the data.

## Discussion

HFS, also known as palmar-plantar erythrodysesthesia (PPE), is a distinctive skin toxicity reaction that can limit dosage with various chemotherapeutic agents (14). It has been demonstrated that 45% of patients with BC and/or ovarian cancer experience HFS following chemotherapy with PLD, with 4%-7% discontinuing treatment due to HFS (15). Another study found that 44.7% of BC patients undergoing chemotherapy with PLD develop grade II or higher HFS (16). The rate of OM was also elevated among patients treated with PLD during BC therapy, although it is often overlooked. OM involves inflammatory and/or ulcerative lesions of the oral mucosal epithelium, typically caused by immune dysfunction, physical and chemical injuries, pathogenic microorganisms, and drugs (17). Indeed, the incidence of OM in advanced BC patients who underwent chemotherapy with PLD was reported at 16% (18). A systematic review found that the incidence of OM in metastatic BC patients receiving PLD chemotherapy varied from 10% to 68%, with both the incidence and severity of OM increasing with each chemotherapy cycle (19). In this study, 34.7% BC patients treated with PLD developed HFS, with grade I-II HFS making up 83.2% of cases, mostly occurring during cycles 2-3. Alternatively, the incidence rate of OM was 13.9%, with the majority being grade I-II, occurring predominantly during the 2nd and 3rd cycles, and then stabilizing after the 3rd cycle. Thus, our findings enhance and build upon previous research.

The incidence rate of HFS is proportional to the dose of PLD (16, 20), and the plasma peak concentration (C_max_) is also reported to be strongly associated with the grade of OM (21). The half-life of PLD is approximately 70 hours. As chemotherapy cycles progress, PLD remains in the body for an extended period, leading to a slow clearance rate and a gradual worsening of HFS and OM, which tends to stabilize after reaching a certain level (22). Interestingly, PLD clearance tends to reach saturation at higher doses. For instance, in Kaposi’s sarcoma, clearance at the standard dose of 40~60 mg/m² is significantly lower than at a reduced dose of 20 mg/m² (23). Here, the dose-dependent increase in HFS and OM incidence (35 vs. 30 mg/m^2^) aligns with PLD’s pharmacokinetics, suggesting cumulative toxicity at higher doses.

A retrospective study found no statistically significant association between the number HFS cases and increasing body mass index (BMI) in patients with current ovarian cancer treated with PLD (24). Nevertheless, some studies have indicated that BMI is an independent risk factor for moderate to severe HFS, with the incidence of these more severe forms rising alongside BMI (20, 25).This discrepancy may result from variations in the types of cancer studied, the sizes and stratification methods of the samples. Despite these findings, the link between BMI and the risk of HFS remains unclear, and this study also did not find a correlation between BMI and HFS.

Just as has been reported, a history of cholelithiasis and elevated levels of ALT, AST, and GGT have been identified as risk factors for HFS in patients with lymphoma undergoing PLD chemotherapy (16). Here, this study also found that elevated ALT and AST, along with a history of cholelithiasis, were associated with an increased risk of HFS. We can examine the factors contributing to the increased incidence of HFS associated with elevated transaminase levels from several perspectives. First, PLD is mainly metabolized by the liver and excreted through bile (26). Therefore, it can be reasonably assumed that impaired liver function or poor bile excretion can lead to PLD accumulation in the body, potentially triggering HFS. Secondly, the liver serves as the primary site for synthesizing antioxidant substances, such as glutathione. When liver injury occurs, its antioxidant capacity diminishes, leading to an increased generation of reactive oxygen species (ROS) by PLD in tissues (27). This process directly damages keratinocytes and vascular endothelial cells, thereby triggering skin inflammation and apoptosis in the hands and feet. Additionally, hepatocyte injury triggers the release of pro-inflammatory factors, such as TNF-αand IL-6, which can intensify systemic inflammatory responses and exacerbate microvascular lesions in the skin, thereby facilitating the development of HFS (28). Some studies revealed that that a decrease in monocyte (MONO) count could speed up the clearance of PLD, thereby reducing the severity and incidence of HFS (29). However, other studies have found no link between MONO count and HFS (30). In this study, MONO count was not associated with HFS. Further investigation into the relationship between MONO count and HFS through prospective and randomized clinical trials is warranted.

In a retrospective study, HER-2 positivity was initially identified as an independent risk factor for HFS, indicating that HER-2+ patients were more susceptible to developing HFS compared to HER-2- patients. However, in this study, no association was found between HER-2 status and the occurrence of HFS. Instead, low Hb levels were identified as a related risk factor for HFS, with patients exhibiting lower Hb more likely to develop the condition. Actually, it has been demonstrated that Hb is associated with skin blood flow, which is significantly reduced as Hb levels decrease (31). Moreover, anemia with low Hb can exacerbate tissue hypoxia and promote the generation of ROS, which may further damage microvessels and consequently lead to the development of HFS (32). Additionally, anemia diminishes the oxygen supply to tissues, rendering the high-metabolism regions of the palms and soles more vulnerable to drug toxicity. Concurrently, it may influence vascular repair by altering erythropoietin (EPO) levels. These mechanisms collectively expedite the onset of HFS (33). Consequently, our research indicated that patients with anemia are at a higher risk of developing hand-foot syndrome, which is also highly plausible from a mechanistic standpoint. However, previous studies did not consider Hb as a factor in their analyses, and the relationship between Hb and HFS requires further validation.

As shown in **Table 7**, the chemotherapy regimen was not statistically significant based on the p value when the comparison of clinical features between HFS group and non-HFS group was performed using the univariate analysis. However, p value here is quite close to the significance (P-value =0.06) which is worth mentioning. It has been identified that sequential administration of dose-dense cyclophosphamide followed by docetaxel for BC patients has an acceptable toxicity profile where HFS was observed (34). In accordance with evidence suggesting that cyclophosphamide may also induce HFS, the primary distinction among the three regimens in our study was the inclusion or exclusion of cyclophosphamide. Consequently, we hypothesize that cyclophosphamide could be a contributing factor to the occurrence of HFS, leading to a P-value approaching 0.5. However, the correlation between cyclophosphamide administration and the occurrence of HFS still needs further research. More details about the correlation between chemotherapy regimen and HFS generation should be also determined.

A meta-analysis identified age as a risk factor for OM, indicating that patients aged ≥ 60 have a 2.75-fold higher risk of developing OM compared to those under 60 years old (35). Similarly, the risk for patients aged ≥ 50 is 1.443 times greater than for those under 50 (36). However, another study investigating OM induced by chemotherapy in malignant tumors did not identify age as a contributing risk factor (37). Similarly, this study did not find age to be a risk factor for OM. However, our findings suggest that a decreased WBC count is a risk factor for OM induced by PLD, likely due to the reduction in immune function associated with lower WBC levels, which in turn increases the risk of OM. We can expand on the potential biological rationale for the observed associations between reduced WBC count and the high incidence rate of OM. First, OM is a common toxic reaction that frequently occurs in cancer patients during chemotherapy, with a low WBC count identified as its risk factor for OM (38). Furthermore, white blood cells particularly neutrophils, serve as the primary defense against pathogens in the oral mucosa. A decrease in their numbers can lead to increased microbial colonization, especially an overgrowth of oral flora such as streptococcus and anaerobic bacteria, which may compromise the integrity of the mucosal barrier (39). Additionally, a reduction in white blood cell count also results in delayed tissue repair due to diminished secretion of growth factors like TGF-β by neutrophils, adversely affecting mucosal healing (40). Consequently, a decline in WBC can precipitate the onset of OM. A low NEUT count is also considered a risk factor for OM post-chemotherapy, as patients with reduced NEUT counts are more likely to develop OM. However, OM tends to improve as NEUT counts recover (41). Conversely, no correlation was observed between a reduced NEUT count and the occurrence of OM.

PLD tends to accumulate in the exocrine glands of the hands and feet and in the oral mucosa, leading to skin toxicity and mucositis (42). Indeed, patients with HFS are at least three times more likely to develop OM than those without HFS. Moreover, there is a statistically significant relationship between HFS and OM, and the incidence rate of OM is significantly higher in patients with HFS, thus making OM a potential predictor of HFS. In this study, the likelihood of OM was found to be higher in patients with HFS. Increased dose intensity, and a history of cholelithiasis was identified as an independent risk factor for the simultaneous occurrence of HFS and OM, likely due to impaired bile excretion which can lead to PLD accumulation in the body, thereby increasing the risk of developing skin and mucosal toxicity. Furthermore, Patients with reduced WBC counts had a higher likelihood of developing HFS and OM, suggesting that diminished hematopoietic function severely suppresses the immune system, thus elevating the risk of these conditions.

Therefore, a thorough understanding of the mechanisms and risk factors associated with HFS and OM, along with the implementation of appropriate preventive and therapeutic measures, can improve the therapeutic effect of PLD while significantly alleviating patients’ discomfort. Currently, patient education emphasizes the importance of keeping the hands and feet moisturized, avoiding irritating substances such as alcohol and strong detergents, and wearing loose-fitting clothing and footwear to help reduce both the incidence and severity of HFS (43). The application of local ice, such as using frozen gloves or socks at -22°C for 15 min before and after PLD treatment, can also effectively reduce drug extravasation (44). Vasoconstriction can be achieved through cryotherapy to prevent OM; for instance, holding ice cubes or sipping ice water for 30 min before and after chemotherapy is beneficial (45). Alternatively, it is essential to maintain good oral hygiene, such as rinsing the mouth with normal saline to keep it clean. Additionally, min dietary modifications are also crucial to reduce the incidence of OM, including avoiding spicy and excessively hot foods while ensuring adequate nutritional support (46).

The role of pharmacological therapy in the prevention and treatment of HFS and OM remains a subject of debate. Currently, it is widely acknowledged that glucocorticoids and amifostine can significantly alleviate HFS; however, their use should be tailored to a reduced dosage based on the treatment regimen (47, 48). Additionally, amifostine, growth factors, and natural remedies such as honey may also contribute to the prevention and management of OM (49). In conclusion, clinical interventions should be selected according to individual patient conditions, as most drug therapies still require further validation.

## Conclusion

In summary, in the retrospective study of PLD treatment for BC, simultaneous incidence rates of HFS and OM were higher, and the incidence rate of OM was higher in patients with HFS. Meanwhile, dose intensity, history of cholelithiasis, ALT, AST and Hb were related risk factors of HFS: reductions of dose intensity and WBC was a related risk factor of OM, and reductions of dose intensity, history of cholelithiasis and WBC was a related risk factor of simultaneous incidence of HFS and OM. We should pay close attention to patients with high risk factors for HFS or OM, ask them in detail whether they have history of cholelithiasis, and evaluate their liver status to provide individualized treatment for these patients.

## Data Availability

The original contributions presented in the study are included in the article/**Supplementary Material**. Further inquiries can be directed to the corresponding author.
