# Supplementary material for: Analysis of risk factors in breast cancer patients with hand-foot syndrome and oral mucositis caused by pegylated liposomal doxorubicin
Source: Front Oncol. 2025 May 22;15:1564681. doi: 10.3389/fonc.2025.1564681 (PMC12137081; doi:10.3389/fonc.2025.1564681)
Supplement: Supplementary file 1 [file Table1.doc]

**Supplementary Table 1**Analysis of** variance inflation factors (VIFs) for all included variables in table 7**

| Variables | VIF |
| --- | --- |
| Age | 1.05 |
| BMI | 1.02 |
| ECOG score | 1.03 |
| Chemotherapy dose intensity | 1.12 |
| Chemotherapy regimens | 1.08 |
| Pathological type | 1.15 |
| Hypertension | 1.04 |
| Diabetes | 1.02 |
| Gallstone | 1.06 |
| Liver cyst | 1.05 |
| ER | 1.07 |
| PR | 1.06 |
| Ki-67 | 1.03 |
| HER-2 | 1.09 |
| Baseline ALT | 1.18 |
| Baseline AST | 1.20 |
| Baseline GGT | 1.10 |
| Baseline TBIL | 1.02 |
| Baseline Cr | 1.03 |
| Baseline MONO | 1.01 |
| Baseline WBC | 1.02 |
| Baseline NEUT | 1.03 |
| Baseline PLT | 1.01 |
| Baseline Hb | 1.04 |

BMI: body mass index; ECOG: Eastern Cooperative Oncology Group; ALT:Alanine aminotransferase; AST:Aspartate aminotransferase; GGT:Gama-glutamyltransferase; TBIL:Total bilirubin; Cr:Creatinine; MONO:Monocytes;WBC:White blood cell; NEUT: Neutrophils; PLT:Platelet; Hemoglobin:Hb
