# Supplementary material for: Analysis of risk factors in breast cancer patients with hand-foot syndrome and oral mucositis caused by pegylated liposomal doxorubicin
Source: Front Oncol. 2025 May 22;15:1564681. doi: 10.3389/fonc.2025.1564681 (PMC12137081; doi:10.3389/fonc.2025.1564681)
Supplement: Supplementary file 2 [file Table2.doc]

**Supplementary Table 2 Analysis of factors associated with HFS occurrence using ALT/AST and Gallstone interaction model**

| **Variables** | **Category/Unit** | **Adjusted OR (95% CI)** | **P value** |
| --- | --- | --- | --- |
| **Age** | Per year | 1.01 (0.98-1.04) | 0.730 |
| **BMI** | ≥25 vs. <25 | 0.99 (0.65-1.52) | 0.954 |
| **ECOG score** | 1 vs. 0 | 1.68 (0.82-3.43) | 0.155 |
| **Chemotherapy dose** | 35 vs. 30 mg/m² | 2.91 (1.86-4.56) | <0.001* |
| **Gallstone** | Yes vs. No | 13.42 (1.62-111.3) | 0.016* |
| **ALT** | Per 10 U/L increase | 1.15 (1.08-1.23) | <0.001* |
| **AST** | Per 10 U/L increase | 1.12 (1.05-1.20) | <0.001* |
| **ALT × Gallstone** | Interaction term | 1.25 (1.06-1.47) | 0.008* |
| **AST × Gallstone** | Interaction term | 1.18 (1.02-1.37) | 0.028* |
| **Baseline Hb** | Per g/dL | 0.98 (0.96-0.99) | 0.007* |

BMI: body mass index; ECOG: Eastern Cooperative Oncology Group; ALT:Alanine aminotransferase; AST:Aspartate aminotransferase; Hemoglobin:Hb
