# Supplementary material for: Analysis of risk factors in breast cancer patients with hand-foot syndrome and oral mucositis caused by pegylated liposomal doxorubicin
Source: Front Oncol. 2025 May 22;15:1564681. doi: 10.3389/fonc.2025.1564681 (PMC12137081; doi:10.3389/fonc.2025.1564681)
Supplement: Supplementary file 3 [file Table3.doc]

| **Supplementary Table 3 Multivariate analysis of factors influencing HFS occurrence**  **with ALT/AST and gallstone history interaction** | | | | | | | |
| --- | --- | --- | --- | --- | --- | --- | --- |
| Parameters | B | S.E | Wald | Sig | Exp (B) | 95% confidence interval for EXP (B) | |
|  |  |  |  |  |  | Lower | Upper |
| Dose intensity | 0.598 | 0.255 | 5.502 | 0.019 | 1.818 | 1.103 | 2.996 |
| History of gallstones | 2.412 | 1.130 | 4.553 | 0.033 | 11.165 | 1.221 | 102.0 |
| Baseline ALT | 0.016 | 0.008 | 4.102 | 0.043 | 1.016 | 1.000 | 1.032 |
| Baseline AST | 0.036 | 0.015 | 5.623 | 0.018 | 1.037 | 1.006 | 1.068 |
| ALT × Gallstones | 0.012 | 0.005 | 5.760 | 0.016 | 1.012 | 1.002 | 1.022 |
| AST × Gallstones | 0.021 | 0.009 | 5.441 | 0.020 | 1.021 | 1.003 | 1.039 |
| Baseline Hb | -0.032 | 0.010 | 10.245 | 0.001 | 0.968 | 0.949 | 0.988 |

ALT:Alanine aminotransferase; AST:Aspartate aminotransferase; Hemoglobin:Hb.The model was validated using the Hosmer-Lemeshow test, which yielded a P-value of 0.62.
